# Baerveldt implant versus trabeculectomy as the first filtering surgery for uncontrolled primary congenital glaucoma: a randomized clinical trial

**DOI:** 10.5935/0004-2749.20200060

**Published:** 2020

**Authors:** Christiane Rolim-de-Moura, Bruno L. B. Esporcatte, Camila F. Netto, Augusto Paranhos Jr.

**Affiliations:** 1 Glaucoma Division, Department of Ophthalmology and Visual Sciences, Escola Paulista de Medicina, Universidade Federal de São Paulo, São Paulo, SP, Brazil

**Keywords:** Primary congenital glaucoma, Glaucoma drainage implants, Trabeculectomy, Mitomycin, Intraocular pressure, Glaucoma congênito primário, Implantes para drenagem de glaucoma, Trabeculectomia, Mitomicina, Pressão intraocular

## Abstract

**Purpose:**

Our initial goal was to compare the efficacy and safety of a glaucoma drainage device and trabeculectomy for children with primary congenital glaucoma after angular surgery failure. However, we discontinued the study due to the rate of complications and wrote this report to describe the results obtained with the two techniques in this particular group.

**Methods:**

This was a parallel, non-masked, controlled trial that included patients aged 0-13 years who had undergone previous trabeculotomy or goniotomy and presented inadequately controlled glaucoma with an intraocular pressure ≥21 mmHg on maximum tolerated medical therapy. We randomized the patients to undergo either placement of a 250-mm^2^ Baerveldt glaucoma implant or mitomycin-augmented trabeculectomy. The main outcome measure was intraocular pressure control. We calculated complete success (without hypotensive ocular medication) and qualified success (with medication) rates. We defined failure as uncontrolled intraocular pressure, presence of serious complications, abnormal increase in ocular dimensions, or confirmed visual acuity decrease.

**Results:**

We studied 13 eyes of 13 children (five in the glaucoma drainage device group; eight in the trabeculectomy group). Both surgical procedures produced a significant intraocular pressure reduction 12 months after intervention from the baseline (tube group, 22.8 ± 5.9 mmHg to 12.20 ± 4.14 mmHg, *p*=0.0113; trabeculectomy group, 23.7 ± 7.3 mmHg to 15.6 ± 5.9 mmHg, *p*=0.0297). None of the patients in the tube group and 37.5% of those in the trabeculectomy group achieved complete success in intraocular pressure control after 12 months of follow-up (*p*=0.928, Chi-square test). Two patients (40%) had serious complications at the time of tube aperture (implant extrusion, retinal detachment).

**Conclusions:**

Both the tube and trabeculectomy groups presented similar intraocular pressure controls, but complete success was more frequent in the trabeculectomy group. Non-valved glaucoma drainage devices caused potentially blinding complications during tube opening. Because of the small sample size, we could not draw conclusions as to the safety data of the studied technique.

## Abstract (pt)

**Objetivo:**

O objetivo inicial era comparar a eficácia e a segurança do implante de drenagem e a trabeculectomia em crianças com glaucoma congênito primário após falência de cirurgia angular. Como o estudo foi descontinuado devido à taxa de complicações, o objetivo deste artigo foi descrever os resultados das duas técnicas neste grupo específico.

**Métodos:**

Ensaio clínico randomizado, não mascarado, incluindo pacientes com idade de 0 a 13 anos previamente submetidos à goniotomia ou trabeculotomia. Os pacientes, que apresentavam glaucoma não controlado com pressão intraocular ≥21 mmHg em terapia medicamentosa máxima, foram randomizados para o implante de drenagem de Baerveldt 250 mm^2^ (Grupo Tubo) ou trabeculectomia com mitomicina (grupo TREC). O principal desfecho avaliado foi o controle da pressão intraocular. Sucesso completo (sem medicação ocular hipotensora) e sucesso qualificado (com medicação) foram descritos. A falência foi baseada na pressão intraocular não controlada, presença de complicações sérias, aumento anormal das dimensões oculares e diminuição confirmada da acuidade visual.

**Resultados:**

Treze olhos de 13 crianças foram estudados (cinco no grupo Tubo e oito no grupo TREC). Ambos os procedimentos reduziram a pressão intraocular em relação às medidas iniciais após 12 meses da intervenção (grupo Tubo 22.8 ± 5.9 mmHg para 12.20 ± 4.14 mmHg, p=0.0113; grupo TREC, 23.7 ± 7.3 mmHg para 15.6 ± 5.9 mmHg, p=0.0297). Nenhum paciente no grupo Tubo e 37.5% do grupo TREC alcançaram o sucesso completo após 12 meses de acompanhamento (p=0.928, teste qui-quadrado). Dois pacientes (40%) apresentaram sérias complicações no momento da abertura do tubo (ex trusão do implante e descolamento de retina).

**Conclusão:**

Os dois grupos estudados apresentaram resultados semelhantes quanto ao controle da pressão intraocular, mas o sucesso completo foi mais frequente no grupo da trabeculectomia. Implantes de drenagem não valvulados podem cursar com potenciais complicações visuais no momento da abertura do tubo. Devido ao pequeno tamanho da amostra, não foi possível determinar quaisquer dados de segurança conclusivos em relação à técnica estudada.

## INTRODUCTION

Childhood glaucoma is an uncommon condition characterized by elevated intraocular pressure (IOP)-related eye damage. The suspicion of glaucoma in a child should give rise to urgent evaluation. Early diagnosis and prompt IOP control are vital to protect vision^(1)^.

Primary congenital glaucoma (PCG), also called isolated trabeculodysgenesis, is the most common type of glaucoma in early infancy^(1,2)^. Angle surgery (goniotomy or trabeculotomy) is widely used as a first-line surgical treatment^(3)^. Although angle surgery can be curative in up to 90% of cases^(3,4)^, in 2014 we observed that responses of 25% of children with PCG under our care were refractory to one or more angular surgeries, probably due to very late diagnosis and difficulty accessing the healthcare system^(5)^.

Mitomycin C (MMC)-augmented trabeculectomy (TRAB) has evolved during the past 15 years to include fornix-based conjunctival dissection, releasable sutures, and additional anti-scarring applications that result in a more posterior aqueous flow and the development of a diffuse posterior bleb^(6,7)^. These changes reduce the incidence of bleb-related problems. Thus, to control fil tra tion and avoid early failure, frequent under-anesthesia exams (UAEs) are performed to remove or loosen releasable sutures and to analyze the need for further anti-scarring and anti-inflammatory drugs^(8)^.

Studies show that glaucoma drainage devices (GDDs) are also a safe option in cases refractory to angular surgeries, and provide reasonable IOP control for years^(9,10)^. These operations have a significant risk of tube-related problems including tube migration or extrusion^(10)^. However, minor postoperative manipulations are usually required to prevent tube failure, which is desirable in uncooperative children^(11)^.

Consequently, no consensus on the optimal surgical treatment after failed angle surgery exists^(3)^. In adults with uncontrolled glaucoma after a failed trabeculectomy or a cataract extraction with IOL implantation, both non-valved GDD and TRAB produced similar IOP reductions after a one-year follow-up^(12)^.

Our initial aim was to compare the efficacy and safety of non-valved GDD (250-mm^2^ Baerveldt implants) with MMC-augmented TRAB to treat children with PCG and uncontrolled IOP receiving maximum tolerated medical therapy after unsuccessful angular operations. Unfortunately, due to two devastating complications in the tube group, we stopped the enrolment. The data we had obtained were insufficient to allow us to conclude on the efficacy and safety of the procedures. Thus, herein we describe the results obtained in these two groups of patients in order to share our valuable experience with other surgeons. All the patients were followed-up for at least one year.

## METHODS

We conducted this prospective parallel controlled study in a CONSORT-compliant manner at a single referral center for tertiary glaucoma patients in Brazil (the Department of Ophthalmology of the Federal University of São Paulo). The Ethical Committee of the Universidade Federal de São Paulo approved this protocol (#1945/11) and informed parental consent was obtained for each enrolled patient before the study.

We randomized the enrolled children for the placement of either a 250-mm^2^ Baerveldt glaucoma implant or a TRAB with MMC. We used a sequential number, opaque, sealed envelope technique for randomization; a study coordinator generated the envelopes^(13)^, and the responsible surgeon assigned the type of intervention at the time of the operation. Neither the patient nor the clinicians were masked to the randomization assignment during the follow-up.

### Eligibility criteria

Eligible patients had a diagnosis of PCG, aged between 0 and 13 years, prior angular surgery (trabeculotomy or goniotomy), IOP ≥21 mmHg with maximum tolerated medical therapy (as measured with a Goldmann tonometer or an Icare rebound tonometer [Icare, Helsinki, Finland] in an outpatient examination and confirmed with a Perkins tonometer in a UAE examination), and ocular growth in the Sampaolesi’s growing curve in the previous months^(14)^ documented by contact ultrasonic biometry. If both eyes in an individual were eligible for the study, we included the first operated eye only.

We excluded children with other causes of childhood glaucoma, such as secondary childhood glaucoma associated with ocular or systemic anomalies; acquired causes of glaucoma such as uveitis, trauma, or intraocular tumors; or any previous intraocular surgery such as cataract surgery. Additionally, we excluded patients with childhood glaucoma without goniodysgenesis that were diagnosed after age of four and those unable to keep scheduled appointments.

### Interventions

Second-year glaucoma fellowship students under supervision of a glaucoma expert performed all surgeries. Surgeons placed a 250-mm^2^ Baerveldt glaucoma implant in the superotemporal quadrant in all eyes that were randomized to the tube group. A fornix-based conjunctival flap was dissected, the superior and temporal rectus were isolated by hooks, and the implant was placed underneath the muscles 10 mm posterior to the limbus and sutured to the sclera with 7-0 silk.

The surgeons occluded the tube completely with 7-0 polyglactin sutures to temporarily restrict aqueous flow through the device until the plate became encapsulated. Then, they trimmed and inserted the tube into the anterior chamber through a 23-gauge needle track, and positioned it away from the corneal endothelium and above the iris. A donor patch of sclera was used to cover the limbal portion of the tube, and the conjunctiva was sutured closed.

Patients randomized to the trabeculectomy group were subjected to a fornix-based opening in the superior region. Surgeons applied sponges soaked with MMC 0.3 mg/mL over the episclera for three minutes and dissected a partial-thickness scleral flap (4 × 4 mm). Next, they performed a paracentesis and put in place an an terior chamber maintainer irrigated with balanced salt solution. A block of limbal tissue was excised from underneath the flap, and an iridectomy was performed. The scleral flap was approximated to its bed with removable or non-removable 10-0 nylon sutures according to the surgeon’s analysis of drainage at that time. Finally, surgeons closed the conjunctiva with an interrupted 10-0 nylon suture and inspected it for aqueous leakage. Subconjunctival injections of a steroid (dexamethasone) and an antibiotic (gentamycin) were administered at the end of the surgical procedure. All eyes were patched overnight.

After surgery, all patients received daily intensive steroid drops (prednisolone acetate 1%) every two hours and were weaned gradually over three to five months according to the degree of conjunctival inflammation. Antibiotic drops (moxifloxacin) four times daily were stopped within two weeks of surgery.

### Follow-up visits and UAEs

We collected baseline demographics and clinical information for enrolled children. Follow-up visits were scheduled at one day and one week postoperatively. UAEs were scheduled for 1, 3, 6, and 12 months after the operations, except for cooperative children (children willing to be helpful with the ophthalmological exams). On day one and week one, we evaluated IOPs using digital tension or a rebound tonometer, where possible based on patient cooperation, and performed biomicroscopy (data not included in statistical analysis).

We performed all scheduled UAEs during mornings, we measured IOPs with a Perkins tonometer and the corneal diameter with calipers; the axial length was measured with ultrasound biometry. We also performed microscopic evaluations of the anterior chamber and fundoscopies.

Visual acuity (VA) was measured at baseline and during scheduled follow-up visits at six and 12 months. We used Teller cards most often; and evoked visual potential or alternatively Snellen tests were used according to the patient’s age and level of cooperation^(15,16)^. We converted all values to the logMAR scale.

Postoperative interventions and surgical complications were documented at each follow-up visit.

### Surgical outcomes

We defined success as an IOP ≤21 mmHg and >5 mmHg, either with hypotensive drops (qualified success) or without glaucoma medication (complete success). Failure was de fined as an IOP outside the success range, or the occurrence of serious complications such as hemorrhagic choroidal detachment, retinal detachment, unstable ocular dimensions (increase in axial length >0.5 mm from the baseline measurement), confirmed VA decrease (any decrease in logMAR at any point of follow-up), loss of light perception, or tube extrusion. We did not consider bleb needling in TRAB as a failure.

### Statistical analysis

**Figure 1:**
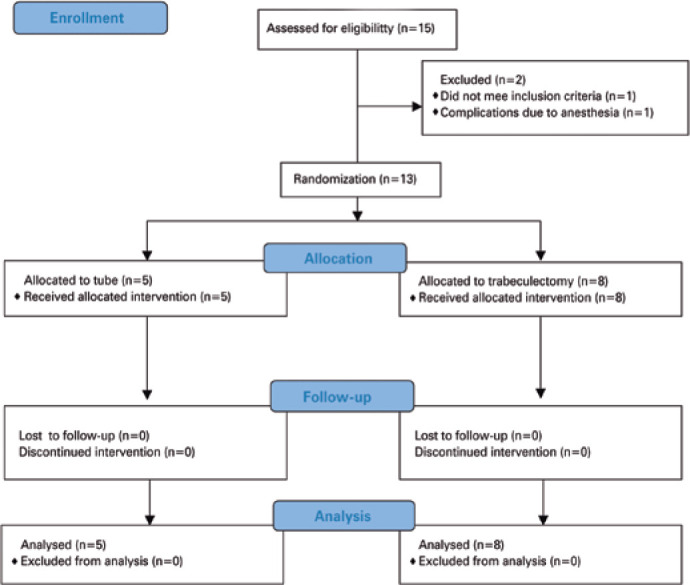
CONSORT Chart: Consolidated Standards of Reporting Trials (Consort) flow diagram of enrolled patients.

We chose eyes, rather than patients, as the unit of analysis due to the marked dependence between eyes. We performed univariate comparisons between treatment groups using a two-sided Student’s *t-*test for continuous variables and Chi-square or Fisher’s exact tests for categorical variables. We converted Snellen VA measurements to logarithms of the minimum angle of resolution (logMAR) equivalents for data analysis. During two consecutive visits, we defined time-to-failure either as the time from surgical treatment to reoperation for glaucoma, or as the time of development of persistent hypotony (IOP <6 mmHg) or inadequately controlled IOP (IOP >21 mmHg). We assessed the risk of treatment failure for statistical significance using the Kaplan-Meier survival analysis log rank test. A *p-*value ≤0.05 was considered significant. Due to a higher rate of complications in the tube group compared with the TRAB group during interim analyses, we interrupted the study. Figure 1 shows the 25 items of the CONSORT 2010 Statement and the study’s flow diagram.

## RESULTS

We enrolled 15 patients in total; two were not randomized to treatment due to one case of anesthetic com plications and one case of an alternative diagnosis observed during UAEs.

We randomized 13 patients to either treatment group, eight to trabeculectomy with MMC (TRAB group) and five to Baerveldt glaucoma implant (tube group). All patients completed at least one year of follow-up.

### Baseline characteristics

Table 1 presents the baseline characteristics of the patients. We found no significant differences in the baseline demographic or clinical features between the groups. Children ranged in age from 10 to 88 months; mean age was 33.31 ± 23.58 months. All eyes had undergone previous angle surgery; mean number of surgeries was 2.4 ± 0.5 in the tube group and 2.5 ± 0.5 in the TRAB group. The preoperative mean number of medications was 2.6 ± 0.5 in both groups. The preoperative IOP between the tube and TRAB groups was similar (22.8 ± 5.9 mmHg vs. 23.7 ± 7.3 mmHg, respectively; *p*=0.580). Both groups presented with similar ocular globe axial enlargements (27.30 ± 1.83 mm vs. 26.27 ± 2.60 mm, respectively; p=0.350).

**Table 1:** Baseline characteristics of patients undergoing Baerveldt implant or trabeculectomy for uncontrolled primary congenital glaucoma (PCG)

	Tube (n=5)	TRAB (n=13)	p-value
Age (months), mean ± SD	40.80 ± 31.66	28.63 ± 17.74	0.109
Gender, n % Male	4 (80)	5 (62.5)	0.201
Female	1 (20)	3 (37.5)	
Race White	5 (100)	7 (87.5)	0.009^*^
Non-white	0	1 (12.5)	
Previous angular surgery, mean ± SD	2.4 ± 0.5	2.5 ± 0.5	0.600
Medication (n), mean ± SD	2.6 ± 0.5	2.6 ± 0.5	0.240
VA logMAR, mean ± SD	1.36 ± 0.79	0.97 ± 0.73	0.550
IOP (mmHg), mean ± SD	22.8 ± 5.9	23.7 ± 7.3	0.580
Corneal diameter (mm), mean ± SD	14.3 ± 0.7	14.7 ± 0.6	0.590
Axial length (mm), mean ± SD	27.30 ± 1.83	26.27 ± 2.60	0.350
C/D ratio, mean ± SD	0.8 ± 0.0	0.8 ± 0.1	0.110

SD= standard deviation; VA= visual acuity; IOP= intraocular pressure; C/D= cup to disc ratio.

We made comparisons between groups using the Student’s *t*-test for continuous variables and the Chi-square or Fisher’s exact tests for categorical variables. A

* indicates a significant difference.

### IOP reduction

Table 2 lists the baseline and follow-up IOPs for the tube and TRAB groups. Both surgical procedures induced a significant IOP reduction. In the tube group, the IOP decreased from 22.8 ± 5.9 mmHg at baseline to 12.20 ± 4.14 mmHg after one year (p=0.0113). In the TRAB group, the IOP decreased from 23.7 ± 7.3 mmHg at baseline to 15.6 ± 5.9 mmHg after one year (p=0.0297). We found no significant difference in mean IOP between the groups at one year (p=0.379), considering all medical and surgical management. However, the TRAB group had significantly lower IOPs than the tube group at the follow-up visits during the first postoperative month (13.6 ± 6.2 mmHg vs. 20.8 ± 18.1 mmHg, respectively; p=0.032).

**Table 2:** Intraocular pressure and medical therapy at baseline and follow-up in patients with uncontrolled PCG who received a Baerveldt implant or trabeculectomy

	Tube (n=5)	TRAB (n=13)	p-value Tube vs TRAB	p-value Tube vs baseline	p-value TRAB vs baseline
Baseline					
lOP (mmHg), mean ± SD	22.8 ± 5.9	23.7 ± 7.3	0.580	-	-
Medication, mean ± SD	2.6 ± 0.5	2.6 ± 0.5	0.240	-	-
1 month					
1OP (mmHg), mean ± SD	20.8 ± 18.1	13.6 ± 6.2	0.032^*^	0.8207	0.0272^*^
Medication, mean ± SD	1.0 ± 1.0	0	<0.001^*^	0.0138^*^	0.056
3 months					
1OP (mmHg), mean ± SD	11.4 ± 6.8	15.0 ± 6.2	0.934	0.0227^*^	0.0224^*^
Medication, mean ± SD	0.4 ± 0.9	0.2 ± 0 .7	0.519	0.0016^*^	0.0271^*^
6 months					
1OP (mmHg), mean ± SD	12.2 ± 6.8	15.1 ± 8.6	0.471	0.0303^*^	0.0494^*^
Medication, mean ± SD	0.8 ± 1.1	0.7 ± 1.2	0.937	0.0111^*^	0.1971^*^
12 months					
1OP (mmHg), mean ± SD	12.20 ± 4.14	15.6 ± 5.9	0.379	0.0113^*^	0.0297^*^
Medication, mean ± SD	0.8 ± 0.8	1.0 ± 1.0	0.233	0.038^*^	0.3547^*^

IOP= intraocular pressure; SD= standard deviation.

The groups were compared using a Student’s *t*-test. A

* indicates a significant difference.

The number of supplementary medications between the tube and TRAB groups at all follow-up visits was similar. A single exception occurred during the first month: the mean number of medications in the tube group was 1.0 ± 1.0 and no patient in the TRAB group was using topical antiglaucomatous drops (p=0.000).

### Surgical outcomes

**Figure 2:**
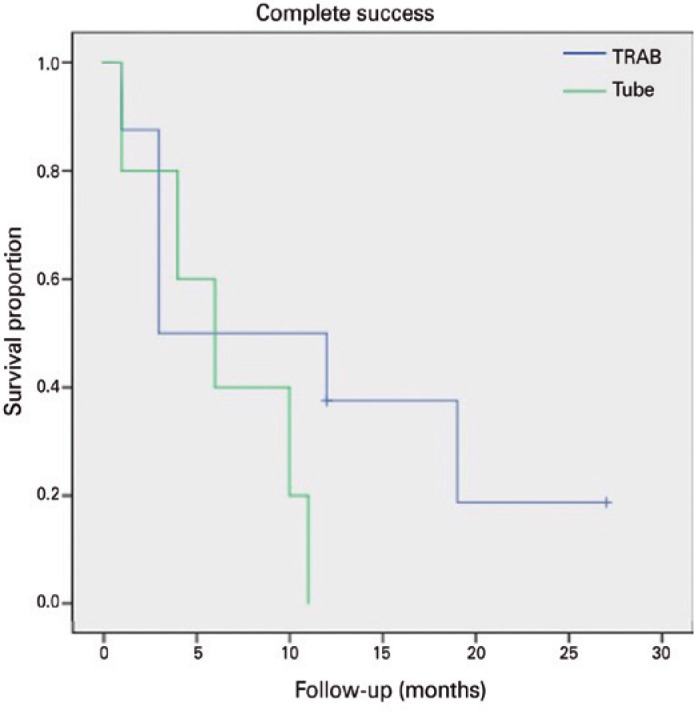
Kaplan-Meier plots of complete surgical success: Kaplan-Meier plots representing the proportion of failure in cases of PCG treated with tube vs. trabeculectomy without any medication.

None of the patients that received a Baerveldt implant had achieved complete success at 12 months. The TRAB group had a complete success rate of 37.5% after 12 months of follow-up (p=0.118, Chi-square test-Table 3). The failure rate with medication was 40% in the tube group (two patients) and 37.5% in the TRAB group (three patients) (p=0.928-Table 3). We applied a Kaplan-Meier survival analysis to compare failure rates between the two groups, as presented in figura 2 (complete success) and 3 (qualified success) (p=0.219 and p=0.856, respectively; log rank test). Figure 4 and table 3 present the failure rates of the two treatment groups due to axial length growths >0.5 mm; we found no significant differen ces between the two groups (p=0.134; log rank test).

**Table 3:** Treatment outcomes during one-year follow-up in patients with uncontrolled PCG who received a Baerveldt implant or trabeculectomy

	Tube (n=5)	TRAB (n=8)	p-value
Failure rate (IOP)			
Without medication	5 (100%)	5 (62.5%)	0.118
With medication	2 (40%)	3 (37.5%)	0.928
Failure rate (increased axial length >0.5 mm)	1 (20%)	5 (62.5%)	0.134

The groups were compared using a Chi-square test.

**Figure 3:**
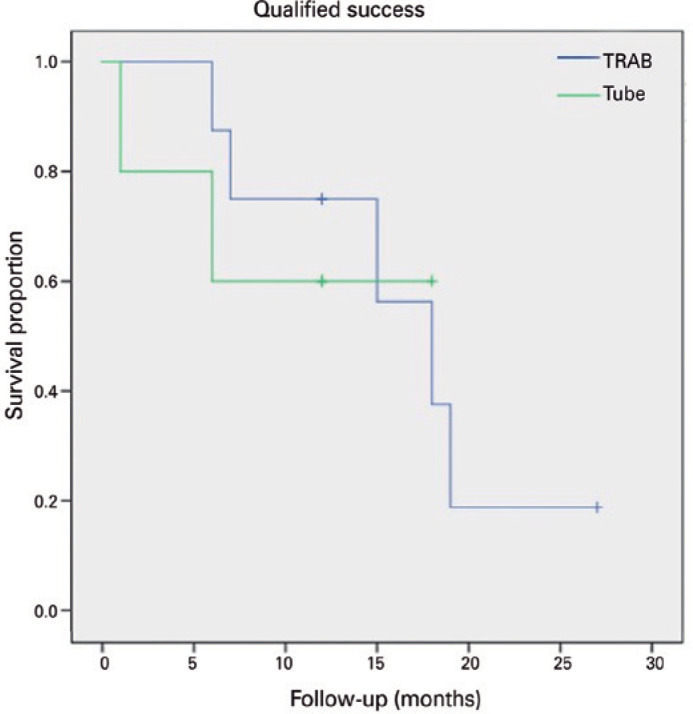
Kaplan-Meier plots of surgical qualified success: Kaplan-Meier plots representing the proportion of failure in cases of primary congenital glaucoma (PCG) treated with tube either trabeculectomy or hypotensive topical medication.

**Figure 4:**
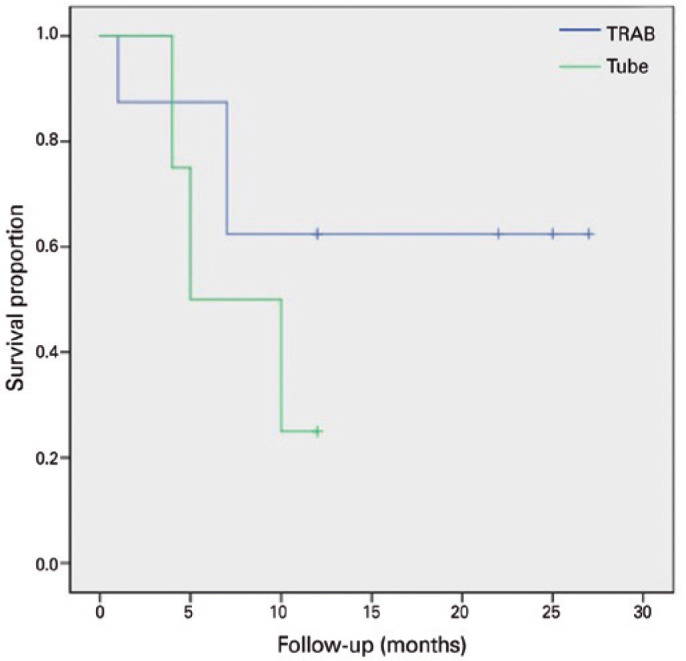
Kaplan-Meier plots of axial length growth >0.5 mm: Kaplan-Meier plots representing the proportions of failure in cases of PCG treated with tube or trabeculectomy

### Surgical complications, reoperations for glaucoma, and other reinterventions

During the first postoperative month, two children (25%) in the TRAB group had shallow anterior chambers and hypotony; as they did not cooperate for tonometry, it was presumed to be due to ocular digital tension. They presented iridocorneal touch in the periphery, but we saw no lens-endothelium touch and no choroidal detachment in the retinal mapping. They were treated with atropine 0.5% twice per day and no surgical intervention was necessary, since the chamber reformed spontaneously. One of these children presented a large bleb and IOP=6 mmHg at the six-month postoperative UAE, but an anterior chamber was formed, and no choroidal detachment was seen. We interpreted the presentation as due to ocular manipulation, and prescribed a plastic patch to wear at night for a month. At a later UAE, the IOP was 8 mmHg and it remained such.

In the tube group, one child presented with low digital tension on the first postoperative day, but due to severe corneal edema we could not measure the anterior chamber depth or see the tube position using biomicroscopy. We found no choroidal detachment on ultrasound images. Digital tension increased on the third postoperative day and we reintroduced hypotensive topical medication. Two patients (40%) had serious complications at the time of tube aperture late in the third postoperative week. One presented with an inferior retinal detachment and the other presented with a flat anterior chamber after extrusion of the plate.

The patients in the TRAB group tended to require more reoperations for glaucoma than those in the tube group, although the difference was not significant. Three patients in the TRAB group (37.5%) required MMC-augmented needling during the 12 months of follow-up. In the tube group, one early repositioning of the implant was necessary as it extruded after one month, and one retinopexy was necessary due to retinal detachment (40% re-intervention rate; p=0.928).

During the second year of follow-up, 50% of the eyes in the TRAB group required further interventions (two Ahmed, one Baerveldt implantation, and one new trabeculectomy), while eyes in the tube group required no interventions (p=0.057).

### VA

We measured VAs of most children using Teller Charts evaluated with visual evoked potentials. The values were all converted to logMAR. We could not measure the VA at baseline in one patient and two patients missed VA measurements at one and six months and at one year because of missed appointments. This group of patients already had a low mean VA logMAR at baseline. Their mean VA was maintained at six months postoperatively, but we observed a decrease at the one-year of follow-up, although it was not significant (baseline, 1.19 ± 0.84; 12 months, 1.28 ± 0.57; p=0.35).

In the eyes of the tube group, the mean VA at the one-year follow-up changed from 1.36 ± 0.79 to 1.47 ± 0.063 (p=0.951). In the TRAB group, the mean VA changed from 0.97 ± 0.73 to 1.04 ± 0.69 (p=0.280). The mean VAs at the one-year follow-up were similar between groups (p=0.951). The VA decreased in three patients of the TRAB group (37.5%) and in three patients of the tube group (60%); but the difference was not significant (p=0.715, Chi-square test).

One patient in the TRAB group (12.5%) and two in the tube group (40%) had improved VAs after one year, probably due to a decrease in corneal edema/opacity.

Two patients, one from the TRAB group (12.5%) and one from the tube group (20%), had hypotony. They later presented with a decrease in VA that was sustained until one year postoperative. One patient in the TRAB group (12.5%) had corneal decompensation, which led to a decrease in VA at one year. Two patients (one from the TRAB group, 12.5%, and one from the tube group, 20%) had a decrease in VA that was probably due to glaucoma progression. One patient in the tube group had a decrease in VA due to retinal detach ment (20%). The causes for these VA decays were similar between groups (Table 4). Table 5 shows all the descriptive data.

**Table 4:** Results of visual acuity in patients who received a Baerveldt implant or trabeculectomy

	Tube (n=5)	TRAB (n=13)	p-value
VA, logMAR mean ± SD			
Baseline	1.36 ± 0.79	0.97 ± 0.73	0.550^*^
1 year	1.47 ± 0.63	1.04 ± 0.69	0.951^*^
Loss of VA, n (%)			
Hypotony	1 (20%)	1 (12.5%)	0.715^†^
Corneal opacity	0	1 (12.5%)	0.410^†^
Glaucoma	3 (60%)	3 (37.5%)	0.428^†^
Retinal detachment	1 (20%)	0	0.187^†^

SD = standard deviation; VA= visual acuity.

The groups were compared using a Student’s *t*-test (^*^) or Chi-square test (^†^).

**Table 5:** Descriptive data of patients

ID	Group	Age (mo)	Operations (n)	VA	1OP (mmHg)	Meds (n)	CD (mm)	AXL (mm)	VA	1OP (mmHg)	Meds (n)	CD (mm)	AXL (mm)	Qualified success	Adverse events
				Preoperative					Postoperative (12 months)						
1	TRAB	15	2	2.8	30	2	15	23.41	1.2	25	3	15	26.43	N	MMC-needling (1 mo)
2	TRAB	55	2	0.18	37	0	14	24.80	0.5	16	0	13	24.68	Y	
3	TRAB	12	3	1.0	18	0	13.5	22.53	1.0	12	0	13.5	22.50	Y	
4	TRAB	14	2	NA	20	3	15	30.60	1.8	17	2	16	33.59	N	Shallow AC (1 day)
5	TRAB	30	3	0.5	15	0	15	27.82	0.7	23	0	15.5	27.50	N	MMC-needling (6 mo)
6	TRAB	40	3	0.3	26	3	15	26.60	0.9	8	0	14.5	23.54	Y	Shallow AC (1 day)
7	TRAB	50	2	0.9	18	3	15	27.13	1.8	14	2	15.5	23.08	Y	MMC-needling (2 mo)
8	TRAB	13	3	1.9	26	2	15	27.28	NA	10	1	15	26.87	Y	
9	TUBE	88	2	1.8	28	3	14	30.45	2.3	6	0	16	NA	N	Retinal detachment (6 mo)
10	TUBE	10	3	0.6	30	2	13.5	26.64	1.0	12	1	15.5	28.69	Y	
11	TUBE	57	2	0.4	20	3	15	27.22	0.9	16	1	13.5	26.03	Y	
12	TUBE	20	2	2.8	16	3	14	25.77	1.98	11	2	14	25.49	Y	
13	TUBE	17	3	1.8	20	2	15	26.43	1.18	16	0	16	27.26	N	Repositioning (4 mo); Implant extrusion (6 mo)

ID= identification; mo= months; VA= visual acuity; IOP= intraocular pressure; Meds= number of medications in use; CD= corneal diameter; AXL, axial length; LP= light perception; NA= not available; Y= yes; N= no; MMC= mitomycin; AC= anterior chamber.

## DISCUSSION

This prospective study enrolled children with uncontrolled PCG who had undergone one, two, or three angular surgeries and were randomized into surgical treatment groups for MMC-augmented TRAB or placement of a 250-mm^2^ Baerveldt implant. All children completed at least one-year follow-ups. TRAB produced an IOP decrea se of 65.8% from the baseline; while tube implantation produced a 53.8% decrease.

Jayaram et al. in their study reported a mean IOP reduction of 45.2% after two years of follow-up in childhood glaucoma patients who underwent TRAB after failed primary goniotomy^(8)^. In contrast to that study, most eyes in our sample did not present an intact conjunctiva due to previous trabeculotomies. In our study, most children were initially staged as advanced cases with cloudy corneas, which made first-line goniotomy impossible. Despite this, we did not observe a significant difference in the mean IOP between the two groups at the one-year follow-up.

The reported success rates with TRAB in childhood glaucoma range from 36% to 95% of cases in retrospective studies^(8,11,17-20)^. Our study was prospective; we observed a qualified success rate of 62.5% after one year of follow-up in the TRAB group.

Retrospective studies of GDD have presented success rates ranging from 44% to 95%^(10,11,21-23)^. However, the etiology of childhood glaucoma and the success criteria varied considerably between these studies. Our qualified success rate after one year of follow-up was 60%.

Beck et al. reported a higher success rate of GDD com pared to TRAB (71.7% vs. 20.8%, respectively), with a mean follow-up period of 15 months. In our randomized clinical trial, the IOP-qualified success criteria were very similar in both groups. The differences in mean IOP and number of medications were seen exclusively in the first postoperative month due to watertight tube ligatures and effective avoidance of initial hypotony in the non-valved GDDs.

However, 37.5% of patients in the TRAB group required an MMC-augmented needling procedure to maintain IOP control during the first year of follow-up. We did not consider this procedure a glaucoma intervention, and we did not classify these eyes as failures. These data were consistent with the retrospective study of Jayaram et al., in which 30% of the patients required bleb needling after TRAB^(8)^.

The eyes of two patients in the tube group experienced early failures, one due to early extrusion requiring tube repositioning and posterior removal, and the other due to retinal detachment followed by retinopexy. Both complications occurred early in the postoperative period after the spontaneous aperture period (spontaneous polyglactin suture release). Patients in the TRAB group did not experience severe complications, four patients required reoperation for IOP control in the second year of follow-up. They also required other antiglaucomatous surgeries in the second year of follow-up, while no eyes in the tube group required further surgeries to control their IOPs.

One patient in each group developed transient late hypotony. In the TRAB group, hypotony, this occurred at the six-month follow-up and we diagnosed the eye as presenting late hyperfiltration that may have been due to ocular manipulation; the patient recovered spontaneously. In the tube group, we detected hypotony after the tube opening coincident with the tube extrusion manipulation.

We observed an early flat anterior chamber in two eyes in the TRAB group (25%), but the patients lacked central athalamia or choroidal effusion, and the complication resolved spontaneously. The rate of anterior flat chamber and choroidal detachment that required viscoelastic injections was 10% ^(8)^. One eye in the tube group presented early hypotony, but no choroidal effusion was diagnosed on ultrasound. On the third postoperative day, the IOP was high and we prescribed antiglaucomatous drops.

We did not observe choroidal effusions, suprachoroidal hemorrhages, retinal detachments, or phthisis in the TRAB group eyes, although these complications have been described in the literature^(11,20)^. Thin, avascular blebs^(17,20)^ were also absent, probably because we used the surgical technique described in the study by Khaw et al. of a fornix-based conjunctival flap, applying mitomycin very posteriorly, and tight-suturing the scleral flap with releasable sutures^(7)^.

However, non-valved GDD surgery complications such as flat anterior chambers and serous choroidal de tachment at the time of tube opening or in the second stage of tube implantation can occur in up to 6% of cases^(10)^. Our patients presented with very advanced buphthalmos that may have led to the posterior retinal detachment (the patient probably had undiagnosed choroidal effusion and choroidal kissing, with posterior retinal detachment) and early plate extrusion. Cataract formation, tube blockage, corneal touch, and strabismus^(10,11,22,23)^ have been described in the literature as complications, but we found none of these in our study.

Tube retractions were also seen in a series with Baerveldt implants for childhood glaucoma treatment^(10,22)^. In our study, children with PCG had a mean age of three years, and we considered ocular growth an outcome and a sign of uncontrolled IOP. Due to our careful observations, only one eye in the tube group (20%) had an axial length increase of 0.5 mm, and it lacked tube retraction.

Although preservation of visual function is the goal of treatment for childhood glaucoma, many factors other than IOP control, such as media clarity, refractive status, and amblyopia treatment influence the outcome. Moreover, VA analysis in these children can be challenging, their cognition increases, and the measurement methods can change during the follow-up periods.

Unfortunately, we still receive children diagnosed with PCG in late stages. In our patients, the mean baseline visual acuity was approximately 20/200 (mean logMAR VA tube group, 1.36 ± 0.79: TRAB group, 0.97 ± 0.73; *p*=0.550). A total of seven eyes had a baseline VA worse than 20/200 (46%), five had VAs better than 20/200 and worse than 20/40 (38.5%), and two had VAs better than 20/40 (15.5%). At the 1-year follow-ups, 8 eyes had 20/200 VA or worse (61.5%), 4 had VAs better than 20/200 and worse than 20/40 (31%), and 1 had a VA better than 20/40 (7.5%). These data differ from those of other studies, which reported that 89% of patients with childhood glaucoma submitted to trabeculectomy had a VA of 20/200 or better after two years of follow-up^(8)^.

We observed no difference in VA between children of the two study groups during all follow-ups. Due to the reserved disease prognosis in advanced childhood glaucoma of our population, we thought maintaining vision better than 20/200 and with no light perception loss one year after intervention in approximately 40% of eyes was reasonable.

This study was limited by its reduced sample size, which made it impossible to detect small differences in IOP measurements and biometry changes. However, the rarity of the disease and the very narrow inclusion criteria made a larger sample size difficult. Moreover, the two early serious complications in the tube group compelled us to discontinue selection of patients for this study.

In all, this study presented a similar rate of success with a similar number of postoperative medications, ocular growth progression, and VA results. Although eyes with TRAB apparently required more surgical interventions to control IOPs, the non-valved GDDs had potentially blinding complications at the tube-opening time, which led us to use this technique sparingly in this group. Because of the small sample size, we did not obtain conclusive safety data regarding the techniques used. We suggest that more studies should be conducted with alternative techniques (such as additional tube ligatures in non-valved GDDs and comparisons with valved GDDs) in representative prospective studies to determine the actual safety of Baerveldt implants to control IOP in buphthalmic eyes.
